# ‘Proto-rivalry’: how the binocular brain identifies gloss

**DOI:** 10.1098/rspb.2016.0383

**Published:** 2016-05-11

**Authors:** Alexander A. Muryy, Roland W. Fleming, Andrew E. Welchman

**Affiliations:** 1 School of Psychology, Highfield Campus, University of Southampton, Southampton SO17 1BJ, UK; 2 Department of Psychology, University of Gießen, Otto-Behaghel-Strasse 10/F, Gießen 35394, Germany; 3 Department of Psychology, University of Cambridge, Downing Street, Cambridge CB2 3EB, UK

**Keywords:** vision, perception, stereopsis, material perception, specularity

## Abstract

Visually identifying glossy surfaces can be crucial for survival (e.g. ice patches on a road), yet estimating gloss is computationally challenging for both human and machine vision. Here, we demonstrate that human gloss perception exploits some surprisingly simple binocular fusion signals, which are likely available early in the visual cortex. In particular, we show that the unusual disparity gradients and vertical offsets produced by reflections create distinctive ‘proto-rivalrous’ (barely fusible) image regions that are a critical indicator of gloss. We find that manipulating the gradients and vertical components of binocular disparities yields predictable changes in material appearance. Removing or occluding proto-rivalrous signals makes surfaces look matte, while artificially adding such signals to images makes them appear glossy. This suggests that the human visual system has internalized the idiosyncratic binocular fusion characteristics of glossy surfaces, providing a straightforward means of estimating surface attributes using low-level image signals.

## 1. Introduction

Material matters. Whether choosing fresh fish, or walking on wet tiles, the visual impression of surface properties influences diverse behaviours. Specularity—the extent to which a surface reflects light like a mirror—conveys important information about an object's physical properties such as its composition, smoothness, and physical state (e.g. wet or dry). However, inferring whether a given surface is glossy or matte is computationally challenging: the image is the result of complex interactions between reflectance properties, three-dimensional shape, and the surrounding illumination. To estimate gloss, the brain must somehow distinguish between reflections, shadows, surface markings, creases, and other features that can produce similar luminance profiles in the image. While disentangling these unknowns is formally intractable, a biological and/or computational solution is likely to be found in the characteristic image features that result from viewing reflective objects. In particular, objects like chrome bumpers or polished kettles create retinal images that are substantially unlike those arising from matte (Lambertian) surfaces. While this idea has a long history [1], we have limited formal understanding of the signals used by the visual system to estimate gloss. Here, we focus on the role played by binocular cues, using a combination of computational analysis and human psychophysics.

Helmholtz [1] noted that while matte surfaces project roughly the same intensity to both eyes, specular surface patches can yield radically different images. In the extreme case of a faceted surface, there can be a complete absence of correspondence between the two eyes' views. Stereograms that present large differences in intensity to the two eyes can lead to an impression of ‘binocular lustre’ [2–4] that is strongest with reversals of contrast [5,6]. However, tests of binocular lustre have been qualitative, with no formal definition of the image quantities measured by the visual system [4,7]. Moreover, rivalrous competition between the two eyes' is often experienced [8], so it remains unclear what role is played by photometric rivalry signals in the perception of surface specularity.

A second potential cue to gloss arises because binocular depth signals differ substantially between matte and glossy surfaces [9–12]. Unlike matte shading or surface markings, specularities ‘float’ some distance in front or behind the physical surface, so the brain might use the offset between specular reflections and the surface to identify glossy materials. Specifically, it was suggested that the brain ‘knows the physics’ of reflections [9], thereby providing a ‘depth offset’ cue indicating that off-surface disparities are caused by specular reflections.

Here, we develop and test the alternative idea that the critical information about gloss relates to the *intrinsic reliabilities* of the disparity signals that are produced when viewing specular objects [13]. In particular, specular objects produce disparities with several unusual properties—including substantial vertical offsets and large disparity gradients—which serve as intrinsic indicators that the depth signals are unreliable. When the brain tries to match specular reflections, many locations on the surface are partially—or only barely—fusible. The result is not complete binocular rivalry, but a discomfiting partial fusion, or ‘proto-rivalry’, which has specific spatial characteristics. We suggest the brain could exploit these low-level binocular signatures to identify specularity based on ‘fusibility’. The merit of this approach is that it captures the generative causes that relate to both (i) photometric rivalry and (ii) depth offsets, potentially providing a unifying account of previous reports from the literature, with a set of image measurements that are likely available at early stages of binocular computation.

**Figure 1.**
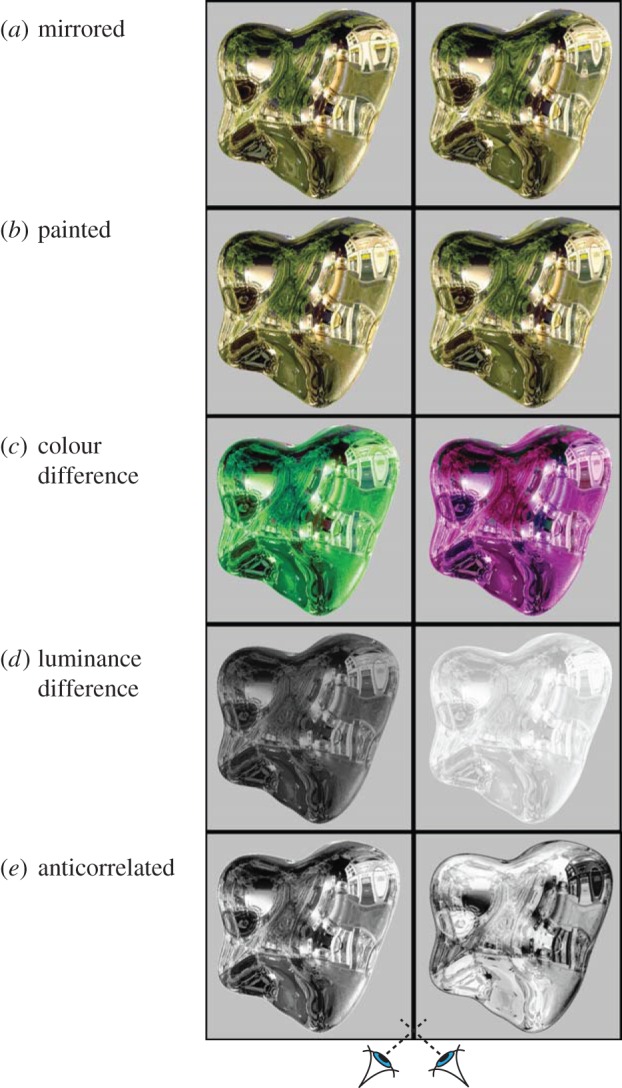
Stereograms (for cross fusion) demonstrating the relative importance of disparity cues and interocular rivalry in gloss perception. (*a*) An ideal mirror ‘potato’ object. (*b*) A ‘painted’ object in which reflections are artificially ‘stuck’ to the surface, like a texture, which removes the disparity offset and rivalrous features. This has a matte appearance. Adding gross interocular differences in colour (*c*) or luminance (*d*) to the ‘painted’ object in (*b*) does not cause it to regain its specular appearance, suggesting such cues are not responsible for the lustrous appearance of (*a*). (*e*) An anti-correlated stereogram in which the intensities of the right image have been inverted. Although this yields a ‘shimmering’ rivalrous percept, most observers report that this is qualitatively different from the impression of a glossy surface in (*a*).

To examine the role of fusibility cues, we rendered stereograms of curved ‘potato’ objects reflecting environments captured from real-world scenes [13] (figure 1*a*). Because the objects were virtual, we could modify the rendering process to ‘paint’ the reflections onto the object, so that—unlike real reflections—they appeared at the same depth as the surface, and are easily fused (figure 1*b*). As such, monocular image properties are practically indistinguishable from those of a specular object (figure 1*a*); importantly, however, when binocularly fused, the object takes on a matte appearance as the reflections appear like surface texture markings (similar to ‘sticky’ reflections based on motion [14]). Differences between the ‘painted’ and ‘specular’ potatoes are perceptually quite apparent (figure 1*a* versus 1*b*), and indicate that binocular signals significantly modify the interpretation of monocular cues. These binocular signals could be due to differences between the ‘painted’ and ‘specular’ shapes in terms of (i) photometric rivalry, (ii) depth offsets, or (iii) fusibility. We therefore set out to test the relative importance of these signals in driving gloss perception.

## 2. Results

To start, we make a brief observation about the general types of image differences that could give rise to an impression of gloss in the light of previous discussions [1,5,6]. In particular, using a ‘painted’ object (figure 1*b*), we made simple image manipulations to induce photometric rivalry in terms of hue, luminance, and contrast (figure 1*c–e*). Viewing these figures suggests that gross forms of photometric rivalry do not induce an impression of material appearance akin to a true specular surface (figure 1*a*). This observation is bolstered by the following formal analysis using systematically controlled binocular stimuli.

**Figure 2.**
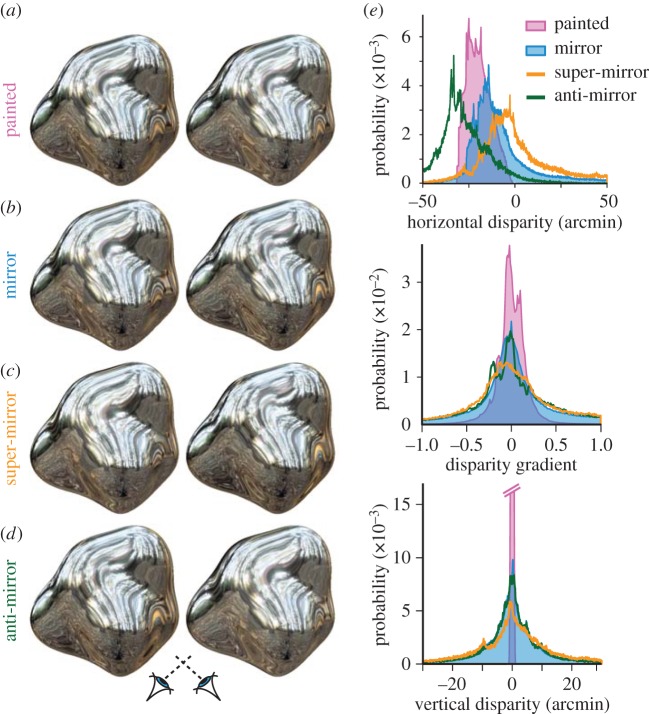
Stereograms (for cross fusion) illustrating ‘potato’ stimuli with different virtual illumination points. (*a*) ‘Painted’: reflections that are ‘stuck’ onto the surface, like texture markings. (*b*) ‘Mirror’: standard rendering of a mirrored surface following the law of specular reflection. (*c*) ‘Super-mirror’: specular reflections are exaggerated, increasing the disparity magnitudes in the stimuli. (*d*) ‘Anti-mirror’: the locations of reflected features are swapped with respect to a true mirror, inverting the disparity sign of the reflections. Most observers report that (*c,d*) look at least as glossy as (*b*), suggesting that physically correct disparities are not necessary for gloss perception. (*e*) Distribution plots of horizontal disparity, disparity gradients, and vertical disparity for the stimuli in (*a*–*d*).

To provide a parametric measurement space for exploring the different cues, we modified the image-rendering process to create objects that lay between ‘painted’ and ‘specular’, and even beyond them. Our goal was to alter disparity properties, while keeping monocular appearance nearly constant (figure 2). To do this, we manipulated the ‘virtual illumination point’ (vIP) for the stereoscopic rendering of the left and right eyes' views of the shapes (see the electronic supplementary material, figure S1 and [15]). We illustrate our approach using four exemplars (figure 2): (i) painted object (vIP = 0) where the illumination effectively acts as a texture stuck to the surface, (ii) mirrored object (vIP = 1) where the illumination follows the physics of specular reflection, (iii) a ‘super-mirror’ (vIP = 2) in which the physical law of reflection is exaggerated, and (iv) an ‘anti-mirror’ (vIP = −1) in which reflected rays for the two eyes are reversed. This manipulation yielded large changes in the patterns of vertical disparities and horizontal disparity gradients in the stimuli (figure 2*e*). While the mirror object appears shiny, and the painted object appears matte (i.e. consistent with the physics of specular and diffuse reflection), the ‘super-mirror’ and ‘anti-mirror’ stimuli also appear shiny, even though their binocularly defined depth structure is very different from what would be created by a real mirrored surface. This already suggests that it is not the specific depths indicated by the disparities that are important for the perception of gloss, but something else about the binocular signals.

**Figure 3.**
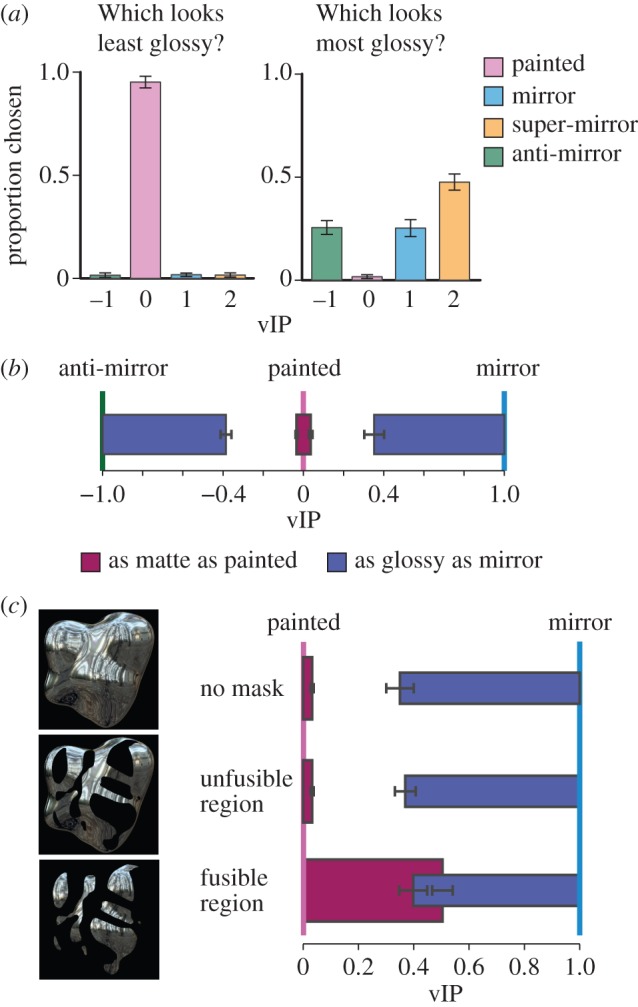
Results of experiments 1–3. (*a*) Proportion of trials on which subjects reported seeing each of the stimuli from figure 2 as ‘least’ (left) and ‘most’ (right) glossy in a direct comparison. (*b*) Range of vIP values that appear at least as glossy as the ‘mirror’ stimulus (blue bars) or as matte as the ‘painted’ stimulus (purple bars). A wide range of both negative (anti-mirror) and positive vIPs appear glossy, whereas only a narrow range close to vIP = 0 appear matte. (*c*) Effects of selectively masking different portions of the object, depending on the local fusibility. When the whole object is shown (top), or only the unfusible portions of the object are visible due to a mask (middle), a wide range of vIPs appear glossy (blue bars) whereas small deviations from the painted stimuli appear matte (purple bars). By contrast, when only the fusible regions of the object are visible (bottom), a much wider range of vIPs appear matte, despite large differences in the disparity values. This suggests that the characteristics of unfusible or partially fusible regions of the image play a key role in gloss perception. Error bars indicate standard error of the mean.

We used these four exemplars to measure the perception of gloss (Experiment 1). Observers (*n* = 13) were presented with four vIP renderings of a ‘potato’ shape, and reported which of the four was the (i) least and (ii) most glossy. When asked to identify the least glossy object, participants readily chose the ‘painted’ stimulus (figure 3*a*). By contrast, their selections for ‘most glossy’ were distributed between the non-zero vIP values: they were greatest for the ‘super-mirror’ condition and similar for the ‘mirror’ and ‘anti-mirror’ conditions (figure 3*a*). This suggests that the physical plausibility of the disparity field is unlikely to be a critical cue to gloss (cf. [12,16]). Rather, it seems participants identify some general binocular image properties that vary with vIP.

**Figure 4.**
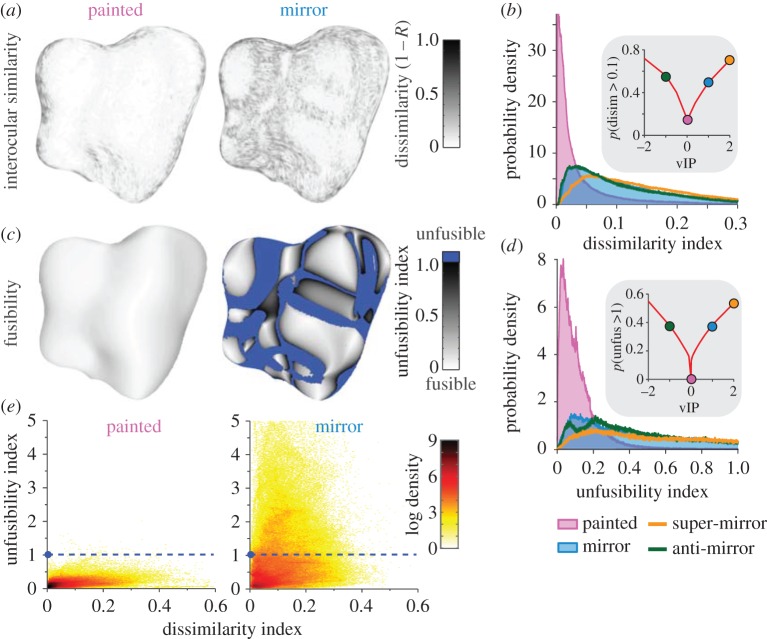
Key characteristics of specular disparities. (*a*) Spatial maps of the interocular image correlation of matched features for ‘painted’ and ‘mirror’ objects (black indicates dissimilar images between the eyes, i.e. low correlation in the binocular matches). (*b*) Distributions of dissimilarity index for the different stimulus types from figure 2. The insert plot shows how dissimilarity varies as a function of vIP. ‘Painted’ stimuli (pink, vIP = 0) represent a sharp local minimum in this function (*c*) spatial maps of the unfusibility index for ‘painted’ and ‘mirror’ objects (white indicates high fusibility; black, poor fusibility; blue, locations that exceed human fusibility limits). (*d*) Distributions of unfusibility index for the different stimulus types from figure 2. The insert plot shows how unfusibility varies as a function of vIP. Again, ‘painted’ stimuli (pink, vIP = 0) represent a sharp local minimum in this function. (*e*) The relationship between dissimilarity and fusibility indices calculated for ‘painted’ and ‘mirror’ versions of three different objects under different illuminations. The logarithm of probability density is plotted on a colour saturation scale. The dashed blued line indicates the cut-off used to classify disparity estimates as unfusible.

To understand these cues, we performed a computational image analysis. To quantify photometric rivalry, we constructed a simple binocular matching algorithm, which computed the image correlation of small square apertures (side length = 6 arcmin) for a range of potential disparities along epipolar lines with some vertical tolerance (±12 arcmin). This approximation of human binocular matching is deliberately simple to avoid the assumptions and parameters required by more advanced algorithms: our goal is to measure potential image information, not to model neural correspondence computations. As a sanity check, we confirmed that this approach could correctly recover the physical surface of the painted object (see Methods and [15] for details). To characterize photometric inconsistencies, we measured the correlation between the left and right eye views (figure 4*a*), parametrized by the Pearson correlation coefficient (*R*). This allows us to quantify, on a continuous scale, the extent to which the matched portions differed interocularly.

Based on previous ideas about photometric rivalry [1,5,6], the visual system could use interocular reversals of contrast to identify shiny surfaces. To provide a useful cue, shiny and matte objects should therefore differ in the degree of *anti-correlation* that they evoke. To determine whether this was true, we quantified matches between the two eyes based on the maximum negative correlation (i.e. the tendency for matching regions to have an opposite contrast sign). This metric allows us to test Helmholtz's hypothesis that binocular lustre is driven by photometric anti-correlation between the two eyes. We found that anti-correlated matches had almost identical distributions for these objects over a wide range of vIPs, suggesting that specular objects generally do not yield systematically more anti-correlation than matte objects. Specifically, we found a very high correlation (*R* = 0.999) between the distributions for the painted (vIP = 0) and mirrored (vIP = 1) objects. Thus, despite its prominence in early work on lustre [1], we can therefore rule out anti-correlation as a strong gloss cue for naturalistic objects.

Nevertheless, it is possible that rather than exhibiting extreme rivalry, images are simply *less* similar for glossy than for matte objects. Considering an individual ‘potato’ object (figure 4*a*), we observe that binocular images are more similar for the painted shape (dissimilarity index distribution peak at 0.003) than for its specular counterpart (peak at 0.035). Although this is an order of magnitude difference, it nevertheless corresponds to an interocular correlation of 0.965; i.e. almost perfect correlation for most locations on specular surfaces. Thus, specular objects appear to give rise to a slight reduction in image similarity, rather than the dramatic absence of correlation suggested by Helmholtz for a faceted surface [1].

We quantified the distributions of interocular similarity (proportion of image locations where the dissimilarity index is more than 0.1) to show how image correlation changes with vIP (figure 4*b*). This identified a clear minimum for painted (vIP = 0) objects, with roughly symmetrical monotonic increases away from zero. Thus, a metric based on interocular correlation could conceivably explain our initial observations that maximum gloss was found at vIP = 2, and that the results for mirror and anti-mirror were comparable. This suggests that rather than dramatic interocular differences, subtle reductions in matching fidelity—as indicated by reductions in interocular correlation—may provide a quantity that predicts when surfaces appear glossy.

## 3. Understanding the generative process

The correlation approach provides a simple image-based clue to changes caused by specular reflection, yet does not explain *why* correlations are reduced. To understand the origin of the photometric cues, we need to consider the generative process. To do this, we calculated binocular correspondence based on matching view vectors from the two eyes (i.e. working out the disparities for which the two eyes saw the same part of the surrounding environment [15]). Using these matches, we measured two properties of the binocular vector fields produced by different vIPs (figure 2*e*): (i) the distributions of horizontal disparity gradients and (ii) the magnitude of vertical disparities (analogous to epipolar deviations in optic flow fields created by specular surfaces, [17,18]). We previously suggested that these binocular measurements may provide important cues to indicate that some depth signals created by specular surfaces are intrinsically unreliable [13]. Here, we tested whether they also play a role in the perception of surface reflectance, by directly indicating the presence of atypical matching caused by specular surfaces.

Changing the vIP from zero (i.e. away from ‘painted’) leads to an increase in extreme disparity gradients (i.e. values that exceed perceptual limits [19]) and a marked change in the vertical disparity structure of the images. By combining measures of these two quantities, we defined a *fusibility metric* [13] for each location on a shape. We find that manipulating vIP causes systematic changes in the areas of a shape which are fusible (figure 4*c*), a behaviour which we captured using the proportion of image pixels that are fusible (figure 4*d*).

It should be noted that in general, fusibility and photometric dissimilarity are related quantities (figure 4*e*). This makes intuitive sense because ray mismatches that reduce fusibility also tend to reduce interocular correlation around matches. Both measures capture in different ways the ‘residual error’ of a match: that is, what is left over, having tried to find the best match. However, fusibility is more directly related to the underlying generative process.

Based on the fusibility metric, we find that even a very slight change in the vIP away from 0—the painted case—causes a big increase in the proportion of unfusible image regions (i.e. a very sharp minimum at zero vIP). As was noted for the correlation statistics, the function monotonically increases away from zero, potentially explaining why mirrors and anti-mirrors have similar judged gloss, while vIP = 2 (super-mirror) is chosen as most glossy slightly more often (figure 3*a*). Taken together, the results of *Experiment 1* and the image analysis suggest that observers rely on the *fusibility* of the stimulus when asked to judge whether a surface is shiny or matte.

In *Experiment 2*, we found that these distinctive V-shaped functions, with pronounced minima centred on the ‘painted’ stimuli, predict how gloss judgements vary as a function of vIP. We instructed participants (*n* = 6) to judge which of two presented stimuli appeared glossier. The target stimulus was either a mirror (vIP = 1) or a painted object (vIP = 0), while the comparison stimulus was chosen from the continuous space of vIP renderings. By adaptively changing the vIP of the comparison stimulus (within the range [−1, 0] or [0, 1]) using a staircase procedure, we identified thresholds (in terms of vIP) for differences in the appearance of mirror and painted objects. We represent these data in terms of the portions of the vIP space that are perceptually indistinguishable from a true mirror or a painted object—i.e. the places for which appearance is judged the same (figure 3*b*). Considering thresholds for painted objects, we find a very small range of vIPs that perceptually match a true painted object. Observers notice very slight perturbations of the disparity field towards a specular object—i.e. matte objects represent a subjective ‘singularity’ in the range of disparity fields. By contrast, thresholds for mirrored objects show that a large region of vIP space is perceptually indistinguishable from a true mirrored object. Moreover, the physical plausibility of the object makes little difference—(unsigned) thresholds are near identical (*F*_1,5_ < 1, *p* = 0.78) in the [0,1] region as in the [−1,0] region. These findings are consistent with the V-shaped functions (figure 4*c*), suggesting that low-level properties of the binocular signals predict participants' judgements.

To test this possibility more directly, in *Experiment 3*, we selectively masked different portions of the shape based on the fusibility index (i.e. less than or greater than or equal to 1), and repeated the measurements (figure 3*c*). First, we consider the data for thresholds for deviations away from the painted shape (figure 3*c*, purple coloured bars). When only unfusible regions are shown, thresholds are similar (*F*_1,5_ < 1, *p* = 0.72) to those obtained for the baseline case where the whole object is shown. By contrast, when fusible regions are presented, the area of perceptual equivalence between a painted object and specular object increased dramatically (*F*_1,5_ = 141.5, *p* < 0.001). This suggests that in the absence of unfusible regions (i.e. due to the mask), it is very difficult to distinguish between shiny and matte surfaces using binocular information, even though the disparity values were far above threshold discriminability. Performance for thresholds relative to the mirrored object (blue bars), did not differ significantly under different masking conditions (*F*_2,10_ < 1, *p* = 0.75), indicating that the changes in the images due to the vIP manipulation produce comparably detectable transitions for both the fusible and unfusible portions of the shapes. Together, these findings suggest that fusibility, rather than depth signals are crucial binocular cues to gloss.

## 4. Making a matte surface appear glossy

**Figure 5.**
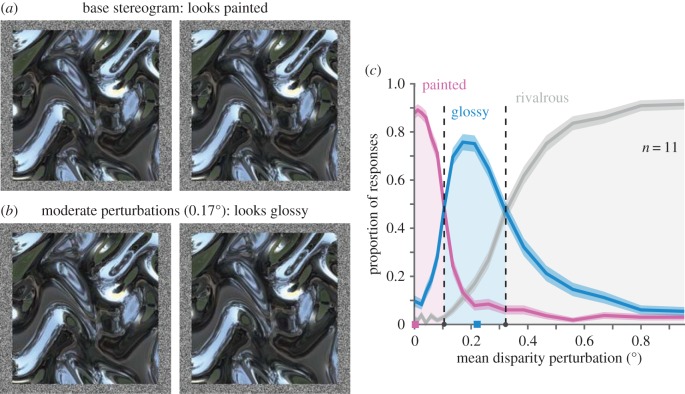
Stimuli and results of Experiment 4. (*a*) The base stereogram (for cross fusion), which was perturbed to create the other stimuli, and elicited a matte painted percept. (*b*) Example of a moderate degree of perturbation (mean 0.17° visual angle), which elicited a glossy percept. In the experiment, the stimuli subtended a 13.6° visual angle, so magnification is required to experience the correct perturbation magnitudes with the images presented here. (*c*) Mean proportion of painted, glossy, and rivalrous responses across all 11 participants, for each of the 18 levels of disparity perturbation tested in the experiment. Margins represent s.e.m. of data pooled across all subjects. Pink and blue squares indicate the perturbation values displayed in (*a*) and (*b*), respectively.

The results discussed so far indicate that low-level properties of binocular signals play a substantial role in perceived gloss. We reasoned that if partially fusible features are crucial for gloss perception, we should be able to induce an impression of gloss by introducing specular-like matching difficulties into an otherwise matte (painted) stereogram. To this end in *Experiment 4*, we created a base stereogram of an irregularly undulating surface with a ‘painted’ appearance (i.e. monocular appearance of a mirror, but with disparities specifying surface markings rather than reflections; figure 5*a*). We then systematically warped these stereograms by applying a spatially varying random distortion function to the monocular images in opposite directions for the left and right eyes' images (figure 5*b*). We reasoned that these warping operations should alter the fusibility of the binocular disparities across the image: in some locations by very little and in others quite considerably. Our aim was to introduce local regions where binocular fusion becomes challenging, akin to the zones of partial-fusion characteristic of specular objects. However, unlike real mirrored surfaces, these locations were not systematically related to either the monocular shape or the baseline disparities of the surface. We parametrically varied the magnitude of the distortions, and asked participants (*n* = 11) to categorize the appearance of each stimulus as ‘matte’, ‘glossy’, or ‘rivalrous’. They were explicitly told that it was not necessary to provide equal numbers of responses for each category or even to use all categories unless they experienced the corresponding percepts.

We found that the perturbations caused substantial and predictable changes in appearance. For small (barely detectable) perturbations, the surface appeared matte (figure 5*d*, pink series). For much larger perturbations—beyond the fusion limits of the visual system—the stimuli appeared rivalrous (figure 5*d*, grey series). However, within a critical range of intermediate perturbations—when the patterns were still fusible, but residual matching errors were clearly noticeable—the surfaces appeared glossy (figure 5*d*, blue series). Further testing (electronic supplementary material, figure S2) suggested that introducing vertical perturbations to disturb epipolar matching is a relatively more potent cue to gloss than horizontal offsets that increase disparity gradients. These results suggest a critical ‘sweet spot’ just below the limits of fusibility that yields an impression of gloss, even when the spatial arrangement of these signals and their depths are inconsistent with the monocularly indicated depth structure.

## 5. Discussion

Identifying gloss is challenging because the retinal images of a surface result from a complex combination of reflectance, illumination, shape, and view geometry. To compute reflectance properties, the brain must somehow disentangle these factors. It has long been thought that binocular vision might provide important signals to achieve this, however, until now it has not been clear which binocular signals are critical. Our findings suggest that rather than using the distinctive depth signals caused by specular reflection [9,10], the visual system appears to have internalized the ‘proto-rivalry’ characteristics associated with shiny surfaces. That is, we find that specular surfaces create subtle departures from fusibility, which the brain exploits as telltale indicators that a surface is shiny, irrespective of the depth structure they convey. This information likely interacts with monocular gloss cues [20–25] to result in perceived appearance.

It is interesting to note that the ‘partial fusibility’ signals we suggest are important for gloss are likely to be present early in visual processing: possibly even at the earliest stages at which binocular matches are computed. This can be contrasted with relatively ‘high-level’ theories of gloss perception based on the deviations of specular reflections from the expected depths of the surface. As long as ‘fusibility’ cues are rarely caused by physical phenomena other than specularity, they provide a reliable, yet easily computed alternative to complex physical computations.

Under specific conditions, large interocular differences in intensity, colour, or contrast can elicit a ‘rivalrous’ impression of lustre. However, we find that curved surfaces under natural illumination conditions do not commonly create such signals, contrary to widely held interpretations of Helmholtz's work. Indeed, we find that there is a specific range—at the fringes of fusibility—where interocular differences yield a percept of glossiness. Below this range, when all signals are fusible, surfaces appear matte. Beyond it—in a range that is unlikely to be created by viewing specular surfaces in the real world—images appear rivalrous. However, within the crucial ‘sweet spot’ of borderline fusibility, the interocular differences are interpreted as arising from surface gloss. Thus, the brain appears to rely much more on subtle, but diagnostic, indicators of specularity than wholesale luminance or contrast incompatibility between the two eyes.

It is important to note that decreases in fusibility associated with specularity are not random but systematically organized. The binocular impression of gloss does not result from arbitrary rivalry between the two eyes, but rather arises due to systematic, and sometimes slight deviations from epipolar geometry, causing many features to be fusible, but with significant spatial error. This effect also reduces the interocular correlation, allowing a second, photometric means for the visual system to track residual matching errors. We have suggested previously [13] that borderline fusibility acts as an important indicator of the intrinsic reliability of disparity signals, allowing the visual system to discount misleading depth signals when judging three-dimensional shape. Here, we find that the same features provide a cue to the surface reflectance properties.

The idea that deviations from epipolar geometry may be important for identifying specular surfaces has been discussed in the context of optic flow, for both human [14] and machine vision systems [17,18]. Given a static scene, stereopsis is formally equivalent to two time points of a translating sensor. However, in biological vision there are important differences between stereopsis and motion parallax, which potentially change the nature of the computations and their implementation in the human visual system. In particular, while for optic flow, the direction of motion is potentially unconstrained, in binocular vision, the two eyes are horizontally separated in the head, which imposes a fixed coordinate frame on correspondence computations. This is reflected in the distribution of binocular neurons' receptive fields [26], and imposes an anisotropy on the vectors that can be fused, which has no equivalent for motion. This has the benefit that binocular epipolar deviations can be directly detected as fusion errors by the very earliest binocular receptive fields [27–29], without having to reconstruct three-dimensional models or identify outlier points in the fundamental matrix [18]. To the best of our knowledge, there is no clear analogue to ‘partial fusion’ or binocular rivalry in motion perception. This suggests that despite the obvious formal connections between motion and stereo, in practice, the detection of specular surfaces in binocular vision may be substantially different from in motion. Informal tests in which we viewed the stimuli from *Experiment 4* sequentially rather than as stereopairs elicited subjective impressions of rigid and non-rigid apparent motion, rather than the distinctive lustre—or rivalry—experienced in the binocular displays.

Finally, it is important to note that partial-fusion signals are unlikely to be sufficient on their own to yield a compelling impression of gloss: the monocular properties of the image must also be consistent with a specular surface. However, a complete absence of these signals (as in the ‘painted’ stimuli) appears to be strong enough evidence that the surface is matte to over-ride monocular cues to gloss, making the reflections appear to be surface markings, painted on the surface. We suggest that it is not consistency between monocular and binocular *depth signals per se*, but rather *the absence of partial-fusion signals* that is critical for making ‘painted’ stimuli appear matte.

In sum, we have identified low-level disparity signals that play an important role in the perception of surface gloss. Manipulating these signals directly changes the perception of surface properties and overrides monocularly available information. These binocular image cues to specularity are best expressed in terms of fusibility and have a corollary in terms of interocular correlation. It is an interesting challenge to understand how the visual system learns to distinguish matching failures that are due to problems with its own correspondence computations from those that are due to specular reflections or refractions.

## 6. Methods

Participants had (corrected-to-) normal visual acuity and stereo vision and were naive to the purposes of the study (except author A.A.M. for Experiment 1). They provided written informed consent.

### (a) Apparatus

Experiments 1–3: stimuli were presented on a two-monitor haploscope in which the two eyes viewed separate displays (ViewSonic FB2100x) via front-surfaced mirrors. The viewing distance was 50 cm and the PC's graphics outputs were controlled by an NVIDIA Quadro FX4400 graphics card. Screen resolution was 1 600 × 1 200 pixels at 100 Hz. The two displays were matched and linearized using photometric measurements. Experiment 4 used a similar system except for the monitors (Dell P190S at 1 280 × 1 024 pixels resolution and 60 Hz), driven by an NVIDIA Quadro NVS290 graphics card and viewing distance of 55 cm.

### (b) Stimuli

Stimuli were created and rendered in MATLAB (The MathWorks, Inc.) following methods described elsewhere [13,15]. Briefly, virtual ‘potato’ objects were created by distorting a sphere (radius = 3 cm) with randomized Gaussian bumps to create regions of convexity and concavity. Virtual objects were rendered using light probe illumination maps [30]. For details of virtual illumination point (vIP) manipulation, see the electronic supplementary material, figure S1.

### (c) Analysis

The correlation-based binocular matching algorithm took greyscale images in spherical coordinates (longitude–latitude) as inputs. It searched for binocular matches by correlating the pixels in a small window (6 arcmin^2^) centred at a given location in the left eye with a sliding window of patches in the right eye. Because specular reflections violate epipolar geometry [15], we considered correspondence for windows centred up to ±12 arcmin from the epiopolar line. We identified matches based on the maximum Pearson correlation (or minimum in the case of anti-correlated matching) between the luminance intensities in the two eyes. To avoid additional parameters, we deliberately kept the model simple and only considered matching windows of fixed size, treating all matches within the matching zone in the right eye as equally valid (i.e. no vignetting away from the epipolar line).

To validate the correlation-based approach, we rendered ‘painted’ stimuli and compared the matching results against ‘ground truth’ stereo-matches calculated geometrically (based on matching reflected ray vectors). The geometrical method was superior due to unlimited spatial accuracy, while the correlation-based method is bound by image resolution. Nevertheless, corresponding matches from the two methods were within ±1 arcmin on 80% of samples. Further details on the methods are provided in [15].

### (d) Procedure

#### (i) Experiment 1: four alternative forced choice glossiness judgements

Thirteen participants were presented with four stimuli (vIP of −1, 0, 1, 2) arranged in a 2 × 2 grid, where spatial organization was randomized across trials. All four stimuli (approx. 7.5° each in diameter, centred approx. 9° apart) related to the same ‘potato’ shape, and were illuminated using the same illumination field. Participants selected (i) the least and (ii) the most glossy shape. Presentation time was unlimited. Three different three-dimensional shapes were presented to the participants under two different illumination fields (pink noise illumination and Debevec's [30] eucalyptus grove). Each image type was presented 15 times to each participant. We found no systematic differences between shapes or illuminations and present results that average over these differences.

#### (ii) Experiments 2 and 3: two alternative forced choice glossiness judgements

Seven participants viewed two objects side by side and were asked to select the glossier. One shape was the test stimulus—either painted (vIP = 0) or mirrored (vIP = 1); the other was the comparison stimulus whose vIP was varied in the range [−1,0] or [0,1] by the QUEST threshold algorithm [31] to identify the 83% threshold. The spatial position of test and comparison stimuli was randomized across trials. Three different irregular ‘potato’ shapes were rendered using the eucalyptus illumination field [30]. One participant could not perform the task reliably (thresholds were near ceiling for the ‘painted’ task, and unmeasureable in the other tasks); their data were therefore excluded.

Experiment 3 used the same method as Experiment 2, but differed in the presented stimuli. In particular, we identified unfusible regions of the shapes and used a mask to isolate them. The fusibility criteria were: |Disparity_vertical_| < 12 arcmin, Disparity_horizontal_| < 30 arcmin, |Disparity_horizontal_ Gradient| < 1. There were three mask conditions: no mask, unfusible regions masked out, inverted mask (figure 3*c*). The fusibility mask was calculated for the mirror (vIP = 1) condition and then used for all the other vIPs in the range [0.1].

#### (iii) Experiment 4: subjective classification of Monge patches

Stimuli were irregular Monge patches created by applying a height field to a plane consisting of approximately 166 K faces. The height field was created in Adobe Photoshop® using ‘Render>Clouds’ to generate a 2 048 × 2 048 pink noise image, which was low-pass filtered, contrast normalized, and then warped using the ‘Distort>Wave’ tool with 5 sine generators (Wavelength: 166–657, Amplitude: 1–162 and ‘Repeat Edge Pixels’ option). This image was down-sampled to 512 × 512 and saved as a 32-bit greyscale image to create smooth surface perturbations when applied to the plane. The resulting mesh was rendered using RADIANCE [32] as an ideal mirror from a fronto-parallel viewpoint centred on the middle of the plane, creating a 512 × 512 monocular image of the surface. This image was then warped in MATLAB to create left and right halves of the stereograms shown to observers.

A ‘base’ stereogram was created by warping the pixels horizontally in opposite directions for left and right images, using the disparities specified by the height field that was used to create the original rendering. This created stereoscopic depth undulations that closely matched the shape depicted by the monocular cues, yielding a ‘painted’ appearance. The other stereograms were created by adding perturbations to the disparity field in horizontal and vertical directions in the image plane. Specifically, two other height field images were created in Photoshop, with the same parameters, but different random seeds (i.e. similar statistics but different shape). The values in these maps were normalized to 18 ranges, spaced logarithmically from 0 to 30 pixels (0.79°), to create a series of perturbation maps with different amplitudes. One map controlled the horizontal components of the distortion applied on top of the base disparities, the other (same amplitude, different pattern) controlled the vertical components. Stimuli subtended 13.6°.

Participants (*n* = 11) were first instructed on the differences between ‘matte’, ‘glossy’, and ‘rivalrous’ appearances using (i) the ‘teapot movie’ [14] and (ii) physically accurate stereo renderings of painted and mirror ‘potato’ stimuli. To explain ‘rivalry’, we used a stereogram of a mirror potato illuminated by two different light probes for the left and right eyes. In the main experiment, the horizontal and vertical perturbation stimuli were each shown 15 times in random order (after a practice run of all stimuli shown once). On each trial, the participants indicated whether the stimulus appeared ‘matte’, ‘shiny’, or ‘rivalrous’. Small, labelled versions of the training stimuli were presented next to the main stimulus to remind observers of the definitions of the three terms.

## Supplementary Material

Supplementary Figures S1 and S2
