# Supplementary material for: ‘Proto-rivalry’: how the binocular brain identifies gloss
Source: Proc Biol Sci. 2016 May 11;283(1830):20160383. doi: 10.1098/rspb.2016.0383 (PMC4874713; doi:10.1098/rspb.2016.0383)
Supplement: Supplementary Figures S1 and S2 [file rspb20160383supp1.pdf]

# Figure S1

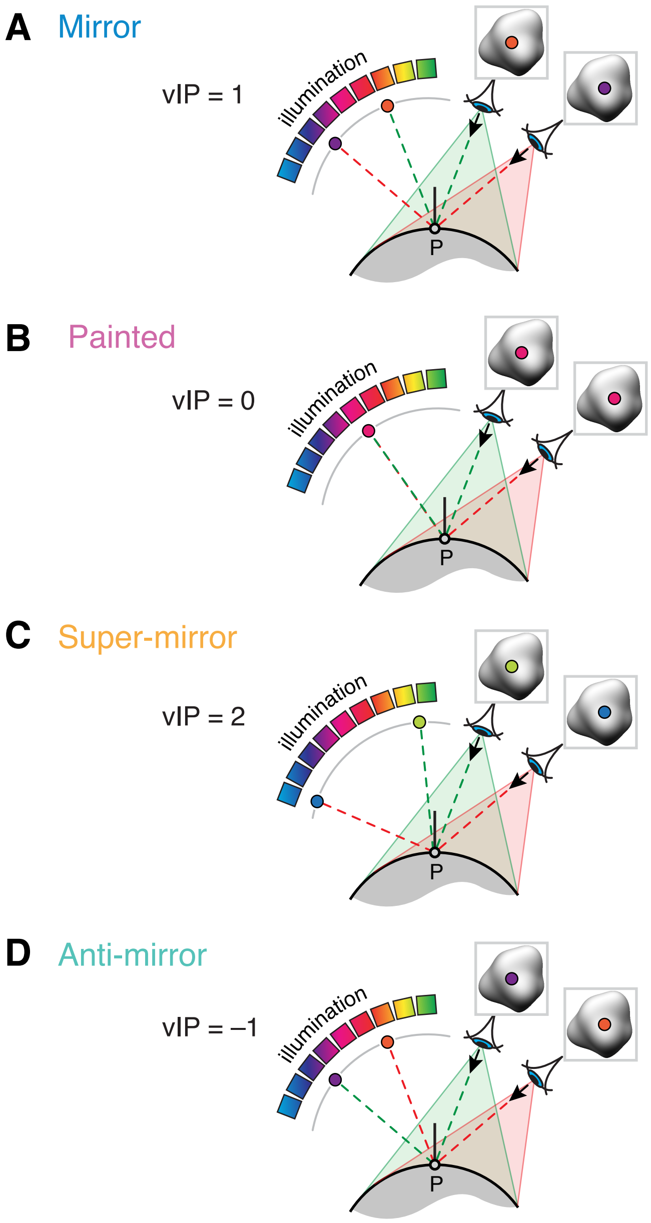


**Figure S1: Illustration of the virtual illumination point (vIP) manipulation.**

The vIP manipulation consisted of changing the mapping between a given location on the object's surface and the pixel intensity of that location as determined by the illumination map. **A**) To start with the simplest case, we describe rendering a true mirror (vIP=1). Here we calculated the ray from the viewer's eye to a given point 'P' on the surface. The appearance of point P was determined by reflecting the view ray around the surface normal (using the physical law of specular reflection) to point into the environment illumination sphere. Doing this for each eye typically results in each eye seeing different portions of the illumination map at point P (depicted using a colour spectrum representation).

To produce different vIPs, we effectively changed the origin of the incoming view ray when calculating the intensity of point P. We did this by virtually translating along the interocular axis while maintaining the correspondence between point P and the retinal position where point P is projected. (Importantly, note that we did not change stereoscopic view frustum with this manipulation, so the pixel projection of surface points remains the unaffected, only their colour value changes). **B**) In the case of a 'painted' object (vIP=0), the colour value at point P was determined by reflecting a view ray around the surface normal where the origin of the view ray for both the left and right eyes was midway between the two eyes on the interocular axis ('the cyclopean point'). This had the result that both eyes see the same part of the illumination map at point P, effectively turning the illumination map into a surface texture. Similarly, a vIP of 0.5 corresponds to view rays for the left and right eyes originating half way between the cyclopean point and the true location of the left and right eyes, while **C**) vIP=2 equates to view rays originating from twice the interocular separation. **D**) For vIP=–1, view rays for the left eye were treated as originating from the location of the right eye and vice versa.

# Figure S2

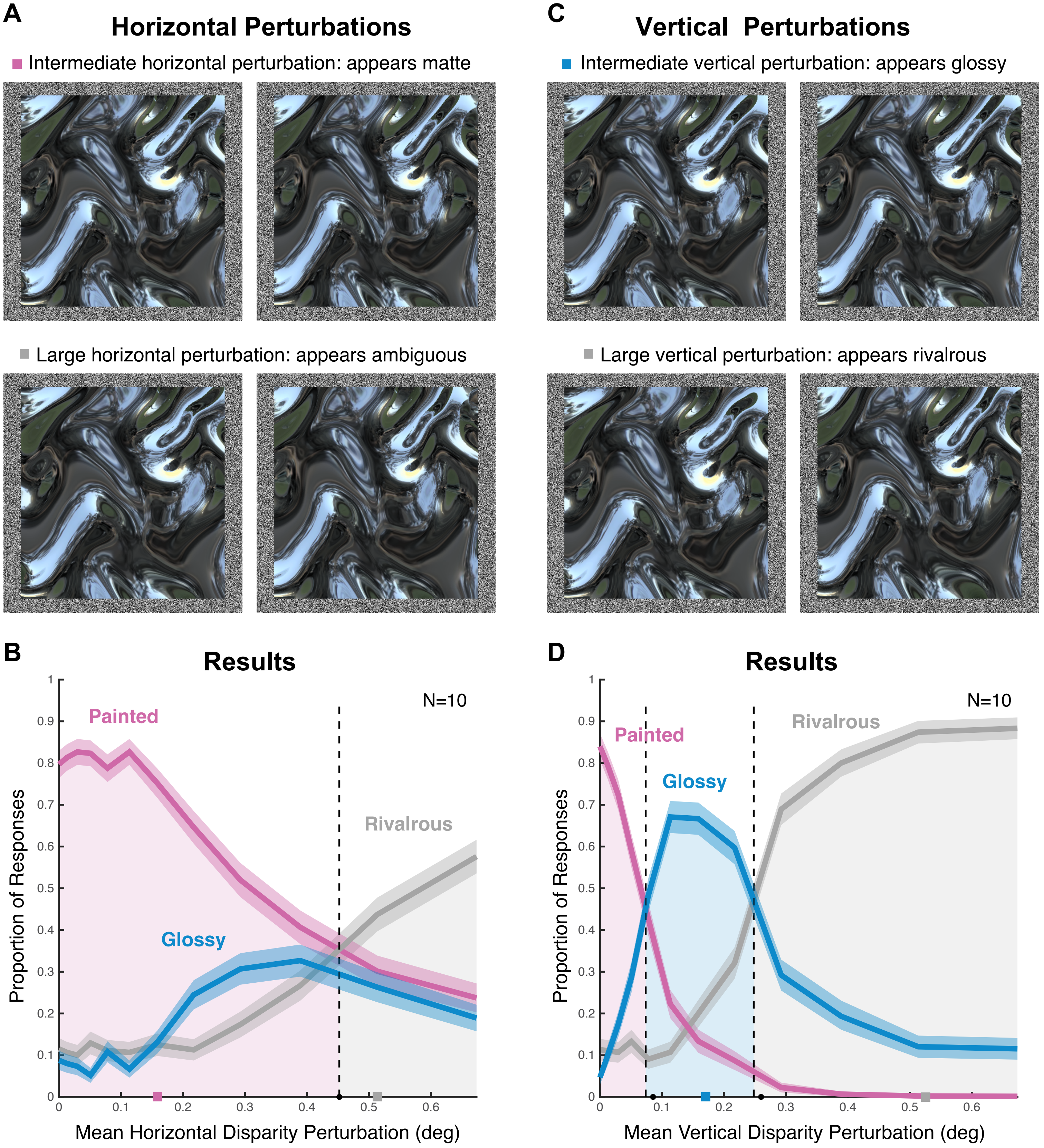


**Figure S2: Effects of horizontal and vertical disparity perturbations**

In Experiment 4 (main text), we applied disparity perturbations to a 'base stereogam' in both horizontal and vertical directions simultaneously. Here we present results of an experiment in which we applied the perturbations in horizontal and vertical directions independently in two sets of stimuli, which were randomly interleved. Ten out of 13 participants passed the screening criterion. All other aspects of the procedure were the same as Experiment 4. (A) Example stereograms for cross fusion, with intermediate (mean 0.16 deg; top row) and large (mean 0.51 deg; bottom row) horizontal perturbations. In the experiment, these stereograms subtended 13.6 deg visul angle, so magnification may be necessary to observe the effects of perturbation correctly. (B) Results for the horizontal perturbations. The stimuli retain a matte appearance over a wide range of perturbations. Only when disparity gradients become large enough to challenge fusion mechanisms do we see a transition to rivalrous interpretations. Five participants exhibited a range where glossy responses dominated, although these were inconsistent across participants. (C) Example stereograms with intermediate (top row) and large (bottom row) vertical perturbations. Note that smaller perturbations yield much stronger changes in the subjective appearance than for the horizontal condition. (D) Results for the vertical perturbations. As in Experiment 4, small perturbations elicit a matte percept, intermediate perturbations yield a glossy percept, and large perturbations yield a rivalrous percept. These results suggest that the results of Experiment 4 are dominated by the effects of vertical perturbations (i.e. non-epipolar disparities).
